# Changing rooms and changing rules: a trans teacher's lessons in gender and institutional ambiguity

**DOI:** 10.3389/fsoc.2025.1656821

**Published:** 2025-09-19

**Authors:** Yi Yau, Ya Chun Shen, Boon Hooi Lim

**Affiliations:** ^1^Department of Leisure and Recreation Management, Taipei City University of Science and Technology, Taipei, Taiwan; ^2^Faculty of Education and Liberal Arts, INTI International University, Nilai, Malaysia

**Keywords:** non-normative embodiment, administrative gender regimes, educational marginalization, gender performativity, transgender teacher

## Abstract

This study adopts a critical autoethnographic approach to explore how a transgender teacher navigates structural violence and institutional erasure during gender transition within Taiwan's educational system, addressing a research gap on transgender educators in Asia. Through embodied narratives of administrative encounters, spatial exclusion, sexual harassment, and pedagogical tensions, the research reveals a disconnect between gender diversity legislation and the institutional inertia of school governance. Drawing on “administrative violence,” “institutional diversity,” and “gender performativity,” it analyzes how everyday practices such as data fields, bathroom access, gendered evaluations, professional recognition, and harassment responses reproduce epistemic violence and marginalization. It further argues that non-normative embodiment, relational pedagogy, and affective labor serve as key strategies for reconfiguring teacher subjectivity and challenging dominant assumptions of a “qualified” educator. By highlighting the dual condition of visibility and vulnerability in classrooms, the research shows transgender teachers as not only victims but also agents of disruption and transformation.

## 1 Introduction

In an era where global awareness of gender equity is steadily rising, the educational field is often envisioned as a key frontier for the realization of diversity and inclusion. However, for transgender teachers, this ideal frequently collides with the realities of institutional constraints. While societies increasingly recognize gender diversity, educational systems largely remain embedded within binary gender frameworks, responding slowly and inadequately to the fluidity and plurality of gender identities (Ingrey, 2013; Woolley, 2015). When a teacher's legal gender is misaligned with their appearance or self-identification, administrative classification becomes ambiguous, further undermining their professional legitimacy and the stability of pedagogical relationships (Harris and Jones, 2014).

In the context of physical education, gender norms are especially pronounced. Teachers are often expected to embody and perform forms of masculinity, with their tone, posture, and bodily comportment subject to student expectations and pedagogical evaluation (Francis, 2008; Chen and Curtner-Smith, 2015; White and Hobson, 2017). Within such a framework, transgender teachers undergoing gender transition experience intensified pressure. They must simultaneously maintain professional authority in the classroom while carefully managing gender expression to avoid exceeding the bounds of institutional tolerance often navigating a precarious zone of being “not too masculine, nor too feminine” (Green et al., 2018).

Although research on transgender teachers has grown over the past decade, particularly within English-speaking contexts such as North America and the UK, offering valuable insights into classroom strategies, teacher-student dynamics, and policy advocacy (Hart and Hart, 2018; Silveira and Goff, 2016; Wells, 2018), the existing literature reveals a noticeable gap in examining the experiences of transgender teachers within Chinese-speaking societies—especially where legal recognition of gender remains inconsistent and administrative systems lack flexible, inclusive gender categories. This study seeks to address that lacuna by providing empirical observation and critical interpretation drawn from the Taiwanese educational context.

Moreover, an additional layer of complexity arises when social class is taken into account. Economic resources and class position shape transgender teachers' capacity to access medical transition, pursue legal recognition, and withstand employment insecurity. Teachers from more privileged backgrounds may leverage professional networks or financial means to navigate institutional hurdles, while those from working-class origins often confront compounded vulnerabilities, including limited access to supportive health services and weaker bargaining power in school governance. This intersection of class and gender identity underscores the need to situate transgender teachers' experiences not only within gendered institutional logics but also within broader socioeconomic hierarchies (Aksoy et al., 2025; Crenshaw, 2013; Luttrell, 1999).

As Sparkes (1994) has noted, teachers often suppress gender differences and opt for “self-silencing” to avoid occupational risks. However, such coping strategies frequently lead to diminished professional confidence and a loss of agency within the classroom. Drawing on my personal experience as a transgender physical education teacher, born in Hong Kong and currently teaching in Taiwan, this study adopts an autoethnographic approach to uncover the institutional constraints, professional negotiations, and pedagogical adjustments encountered during gender transition.

This research focuses not only on gender performance and interactional strategies in classroom settings but also interrogates how institutional mechanisms such as administrative forms, spatial access, and identity verification shape and constrain the professional practice and positionality of transgender teachers. Theoretically, this study integrates Butler's (2002) concept of gender performativity to elucidate how gender expression is simultaneously regulated by cultural norms and institutional logics. Additionally, Spade's (2015) framework of administrative violence is employed to examine how seemingly neutral classification systems produce structural exclusion and marginalization. Spade defines administrative violence as the systemic harm enacted through bureaucratic procedures—such as forms, policies, and eligibility criteria—that disproportionately disadvantage those whose identities fall outside normative gender frameworks.

This article also draws on Pinder and Harlos's (2001) notion of institutional silence, emphasizing how the absence of institutional recognition for non-normative gender identities renders individuals excluded not only from policy protections but also from the discursive frameworks of visibility and intelligibility (Ferfolja, 2005). Moreover, the study is informed by queer pedagogy (Bryson and De Castell, 1993; Nemi Neto, 2018) and trans pedagogy (Keenan, 2017), which view education as a critical site for deconstructing gender norms, fostering dialogue, and enacting transformative practices.

Beyond supplementing the predominantly Western-centric perspective in existing research, this study offers a situated narrative grounded in the gender policy discrepancies between Taiwan and Hong Kong, both embedded in Chinese-speaking cultural contexts. Drawing on Ahmed's (2012) notion of affective politics and Butler's theory of identity negotiation, this research articulates the temporal disjuncture between legal recognition and social practice during gender transition, contributing to a theoretical understanding of liminality and professional ethics in educational contexts.

Recent scholarship has noted the lack of LGBTIQ+ research and curricular integration in the Asia-Pacific educational landscape (Gates et al., 2024). Only a handful of studies have examined transgender teachers in this region. Oculares and Trakulkasemsuk (2025) traced the identity formation of a Filipino transwoman EFL teacher in Thailand, while Lozada et al. (2024) explored the inclusion of transgender women teachers in the Philippines. Importantly, existing scholarship has concentrated largely on male-to-female (MTF) teachers, reflecting the perception that transwomen face sharper societal scrutiny, while female-to-male (FTM) teachers remain comparatively underexplored.

This imbalance underscores both the scarcity and asymmetry of current research. By centering on the experiences of an FTM physical education teacher, this study not only addresses the neglect of FTM perspectives but also foregrounds the structural dimensions of institutional violence, administrative classification, and gendered pedagogical expectations within Chinese-speaking educational contexts. In doing so, it positions itself as a timely intervention, contributing empirical insights as well as region-specific knowledge production.

Based on teaching notes and reflective writings collected between 2021 and 2025, this study employs thematic analysis to identify five core experiential themes: (1) administrative misalignment in gender markers and class rosters, (2) spatial exclusion in restroom and sports facility access, (3) employment challenges due to discrepancies between appearance and legal identity, (4) gaps between institutional protection and actual responses to sexual harassment, and (5) processes of rebuilding trust in teacher-student relationships. These experiences highlight not only the limitations of institutional structures but also illustrate how transgender teachers develop responsive professional identities and pedagogical ethics through practice and reflection.

In sum, this research seeks to bridge the gap in understanding the institutional experiences of transgender teachers in Chinese-language educational contexts. It underscores the dual role of teachers as both knowledge practitioners and institutional negotiators. By integrating empirical insights with critical theory, this study amplifies the voices of transgender educators while addressing the insufficiencies of current gender equity policies in practice (Davis and Yeung, 2022). Ultimately, it offers empirically grounded and critically informed recommendations for the inclusive design of educational systems and the formulation of gender-responsive policy frameworks.

## 2 Materials and methods

This study adopts an autoethnographic approach, drawing on the researcher's lived experience as a transgender teacher to explore the processes of institutional exclusion, spatial constraints, gender negotiation, and professional identity construction within the educational context of Taiwan. Autoethnography, as a qualitative research method that integrates autobiographical narrative and ethnographic inquiry, seeks to illuminate the structural power of cultural institutions through personal experience, while fostering social understanding and critical reflection (Pitard, 2019). The central research question guiding this study is: How do transgender teachers, situated at the intersection of educational institutions and cultural gender norms, navigate the disjuncture between institutional misrecognition and societal expectations to practice professionalism, negotiate identity, and reframe experience?

### 2.1 Researcher positionality and background

The researcher is a transgender man born in Hong Kong and currently teaching at a university in Taiwan. Due to the high cost of gender-affirming surgeries and the impact of the COVID-19 pandemic, his transition has spanned over a decade and remains medically incomplete. Although he has legally changed his gender marker on his British Overseas passport and obtained medical certification in Hong Kong permitting access to male-designated spaces, Taiwan's legal and medical frameworks remain inconsistent. Despite receiving hormone prescriptions based on a diagnosis of gender dysphoria from Taiwanese doctors, the absence of a formal diagnostic certificate precludes legal gender change on the national ID card, thus disqualifying him from legal access to male restrooms and related gendered spaces. This disjunction between medical recognition and administrative practice positions the researcher in a constant state of contradiction and constraint, caught between institutional classifications and everyday spatial use. Such a cross-jurisdictional fracture of gender identity forms the basis of multiple structural challenges faced within the Taiwanese educational environment.

While this autoethnography focuses on a single case, the case itself situated within conflicting transnational legal frameworks, educational systems, and gender classification practices offers a valuable lens for examining how transgender teachers construct identity and negotiate institutional space in the interstices of policy and practice. The researcher has taught physical education and leisure-related courses at various universities, as well as community-based Pilates and exercise classes. These teaching contexts span both on-campus and off-campus sites, full-time and adjunct positions, and a range of student age groups, offering a rich empirical foundation for exploring the visible and invisible processes of exclusion, and the gendered negotiations embedded in different institutional settings.

### 2.2 Data sources and organization

The data for this study are drawn from the first author's firsthand experiences as a transgender teacher in Taiwan between 2021 and 2025. These data are categorized into three types: (1) field notes and post-class reflections that documented classroom interactions, pedagogical adjustments, and gender negotiation; (2) administrative documents and spatial usage records that captured exclusionary practices in gender markers, student rosters, and spatial demarcations; (3) experiences of employment rejection and reimbursement obstacles in off-campus teaching due to mismatches between legal identification and gender presentation.

To enhance analytical consistency and trustworthiness, the second author—a master's student with formal training in qualitative research—assisted in the organization and thematic analysis of the data. Theme coding was conducted through iterative discussions and cross-checking between the two authors, ensuring interpretive triangulation. Five core themes were identified, illustrating how transgender teachers navigate identity negotiation and professional practices within institutional contexts. While the first author maintained the primary narrative role through reflective writing and theoretical engagement, the second author contributed structural insights and ensured coherence and analytic depth. This collaborative approach strengthened both the internal consistency and external credibility of the analysis, aligning with the standards of autoethnographic inquiry that value multi-perspective validation.

### 2.3 Trustworthiness and validity

To ensure the credibility and validity of this qualitative autoethnographic study, we adopted Tracy's (2010) framework of five quality criteria: (1) Situational transparency: the first author's gender identity, legal status, and teaching context are clearly articulated to help readers grasp the positionality of the research; (2) Thick description: concrete scenes, classroom dialogues, and institutional interactions are vividly portrayed to evoke contextual resonance and portray lived realities; (3) Researcher reflexivity: the first author acknowledges internal tensions and emotional contradictions under structural and cultural constraints, revealing the dual positionality of narrator and analyst; (4) Theoretical resonance: critical theories are employed in dialogue with empirical data to deepen analytical meaning; (5) Ethical sensitivity: all narratives are grounded in the first author's personal experiences, and all references to other individuals have been carefully anonymized to protect their privacy and safety.

In order to enhance transparency and analytic rigor, the first author's reflective teaching journals were systematically collected over four academic years (2021–2025) through an ongoing documentation process. After each class session, the author completed a structured reflection form comprising three components: (1) classroom incidents related to gender expression or student reactions, (2) pedagogical strategies and emotional responses, and (3) institutional or administrative interactions. These entries were time-stamped, stored digitally by semester, and periodically reviewed to identify emerging patterns.

During the thematic analysis phase, the second author assisted in coding these reflections using Microsoft Excel. Although not a specialized qualitative software, Excel allowed for structured data entry, customizable coding matrices, and traceable color-coded themes. An initial open coding process was conducted to identify recurring themes, followed by axial coding to cluster the data into conceptually coherent categories. Coding consistency was enhanced through intercoder dialogue and negotiated consensus. Furthermore, reflective notes were cross-referenced with administrative documents and classroom materials to support triangulation and minimize interpretive bias.

Beyond reviewing data categorization, the second author also functioned as a peer debriefer, offering critical feedback and alternative interpretations during the analytic process. This dialogic validation further strengthened reflexivity and analytic transparency, thereby enhancing both the confirmability and credibility of the findings. In addition, the second author challenged interpretive assumptions and jointly validated thematic coherence, reinforcing the overall trustworthiness of the study.

Overall, this research emphasizes both emotional authenticity and cultural interpretation, fulfilling the dual imperative of autoethnography: to remain faithful to lived experience while engaging in critical social analysis.

## 3 Results

### 3.1 Ambiguity and misalignment within the administrative system: when the institution cannot name me

This narrative episode was thematically categorized under the major theme “Ambiguity and Misalignment within the Administrative System.” It illustrates how bureaucratic silence and rigid procedures generate structural misrecognition for transgender educators in Taiwan. Drawing from Spade's (2015) concept of administrative violence and Ahmed's (2012) critique of non-performative institutional inclusion, this section connects personal narrative with broader institutional mechanisms of exclusion.

Analytically, this theme was further divided into two sub-codes: “administrative silence and institutional misrecognition” and “invisibility through documentation.” The first captures how avoidance, delay, or non-action by administrators becomes a recurring mechanism of trans-erasure. The second highlights how rigid forms, records, and bureaucratic classifications function as technologies of exclusion, producing institutional outing, and professional withdrawal.

#### 3.1.1 Administrative silence and institutional misrecognition

In Taiwan's educational system, the legalization of same-sex marriage has not been matched by equivalent advancements in public understanding of transgender identities. This tension between progressive legislation and persistent cultural conservatism manifests in schools as a structural ambiguity and misalignment. School administrators, often not acting out of malice, choose to avoid engagement simply because they “don't know what to do,” thereby perpetuating institutional silence.

For example, when a transgender teacher requests to use a bathroom aligning with their gender identity, administrators may delay decisions, redirect responsibility, or respond with procedural inaction—effectively avoiding confrontation rather than addressing the issue directly. *In my reflective notes, I once wrote: “The silence is louder than rejection; they pretend not to see me, so they don't have to decide where I belong.”* This moment of avoidance, when coded and compared with other similar episodes, consistently pointed to a pattern of administrative non-action as a mechanism of disavowal—one that reinforces institutional trans-erasure through inaction rather than explicit denial.

As Ahmed (2012) argues, institutional diversity slogans frequently become non-performative speech acts: they claim inclusivity but fail in implementation, reducing inclusion to an empty symbol. During my gender transition, although my appearance and voice had already masculinized and most colleagues recognized me as a male teacher, administrative procedures remained strictly tied to the gender listed on my national ID card. *Another entry from my journal captured my frustration: “Why is it that everyone can call me a male teacher, but the system insists I stay a ‘female' forever?”*

#### 3.1.2 Invisibility through documentation

All official forms continued to offer only two binary gender options “male” or “female,” leaving no space for lived experiences that fall in between. When teacher attendance sheets, faculty rosters, public announcements, or sign-in systems repeatedly labeled me as “female,” the experience of being silently outed by the system rendered me a kind of bureaucratic anomaly.

This was not a voluntary disclosure of identity but a form of involuntary exposure, institutionally imposed, stripping me of agency and dignity in how I define myself. “I can allow my name to be listed but why must my gender be?” This question lingered repeatedly in my thoughts. The system made me visible yet refused me autonomy. *In one journal entry, I wrote: “Every time the roster marks me as female, it feels like a forced announcement to everyone that I am not who I say I am.”*

Through iterative coding of my reflective journals and analytic memos, this theme emerged as a repeated site of conflict: the tension between lived identity and institutional classification. When information sovereignty is governed by administrative protocols and technological systems, gender ceases to be a self-defined process and is instead reduced to a passive attribute encoded in a database.

As Spade (2015) critiques, “Administrative violence is not always driven by hostility; rather, it emerges through technical classifications and rule-making that render certain identities unlivable within institutional systems.” These seemingly neutral procedures are in fact mechanisms of institutional outing. Mangin et al. (2022) have noted that transgender teachers are often placed in high-risk scenarios of involuntary disclosure, and such exposure does not always require spoken language.

When standardized forms do not allow flexible gender options, the implicit norms embedded in such designs establish the boundaries of who is allowed to be seen and who must remain hidden, thus reinforcing the legitimacy of institutional exclusion. This is a form of epistemic violence—for example, when gender identities that fall outside the binary are excluded from official forms, the system effectively denies the legitimacy of those lived realities.

This form of administrative misalignment is not confined to the school campus. It resurfaced when I was invited to teach a Pilates course at a long-term care facility. After a well-received session during which the director herself enthusiastically participated and expressed willingness for future collaboration, I submitted a copy of my national ID to complete the hiring process. Immediately, the previously warm response turned silent. Not long after, I was informed by a staff member: “They no longer need this instructor.” No formal explanation was given.

In line with prior narrative themes, this event was coded under “invisibility through documentation,” where a single unchecked box triggered institutional withdrawal—despite positive pedagogical performance. I understood clearly that the issue was not my qualifications or teaching ability, but the gender designation on that document—a singular field that created a rupture in perception. *As I noted in my reflective diary: “It wasn't my teaching they rejected—it was a single box on a form that erased everything I had proven in the classroom.”*

Hazeldean (2019) argues that when bureaucratic systems elevate “data consistency” to the ultimate standard of legitimacy, information that appears objective instead becomes an exclusionary tool. For transgender professionals, this turns documents into sites where social stigma and institutional gatekeeping are enacted, restricting their ability to participate fully in professional life—even beyond the school context.

Spade (2015) further argues that today's most potent tools of exclusion are no longer legal sanctions or punishments, but rather seemingly impartial files, data systems, and identity verification procedures. Though intangible, these institutional techniques operate with precision, quietly determining who may access the system and who must remain outside its gates.

#### 3.1.3 Summary

This experience is hardly new to me, but what struck me was that this time, exclusion did not come from students or administrators, it came from a document, a checkbox, and a form of silent but decisive cancellation. My teaching ability had not changed, and student feedback remained positive. Yet, the institution terminated our collaboration the moment it discovered I was not “the kind of teacher it had assumed.”

The recurrence of these moments across institutional settings further reinforces the analytical claim: administrative misalignment is not an isolated incident but a systemic mode of exclusion. It is precisely through such mundane administrative practices that transgender individuals are continually reminded: you may be utilized, but you are never fully accepted.

### 3.2 Gendered spatial exclusion and silent negotiation

In my career as a physical education teacher, the most persistent challenges have not stemmed from student resistance, but rather from the silent yet deeply entrenched mechanisms of exclusion embedded in the spatial structure of the campus. These exclusions are not necessarily driven by malice; rather, they manifest through the default configurations of spatial design and the tacit norms of institutional classification, requiring gender non-conforming individuals to engage in continual self-negotiation in their everyday practices.

Analytically, this set of narratives was coded under the theme “gendered spatial exclusion,” with sub-codes such as “navigating binary facilities,” “exceptional alternatives,” and “bodily withdrawal.” These categories emerged inductively during open coding of reflective journals and were later clustered to illustrate how spatial structures discipline transgender teachers' daily practices.

#### 3.2.1 Navigating binary facilities

Take swimming classes, for example. To access the pool, instructors must pass through gender-segregated changing rooms and restrooms, which are always marked with only two options: “male” and “female.” While my appearance and voice had already masculinized and students and colleagues routinely referred to me as a “male teacher,” the unchanged gender marker on my national ID meant I was not institutionally recognized as male. Even after proactively informing the school about my gender transition, the administration never clearly explained how I should navigate these gender-segregated spaces. The issue was never my ability to teach swimming but rather that, before any lesson could begin, I was forced to confront a clash between spatial governance and gender identity. *As I wrote in my reflective journal: “Standing in front of two doors marked male and female, I realized that no matter which one I chose, I would be questioned.”*

#### 3.2.2 Exceptional alternatives

The fitness center presented similar challenges. While the main entrance was not gendered, the facilities for showering, changing, and toileting remained strictly binary. Some suggested I use the accessible restroom, but this was never a true third option, it was an exceptional “alternative space” whose access still required passage through the male/female binary corridor. Such a spatial arrangement did not signify inclusion, but rather temporary “tolerance,” wherein I became silently categorized as an administrative anomaly, subjected to exceptional handling. *In my notes, I once wrote: “Walking toward the so-called ‘accessible' restroom felt less like accommodation and more like being sent to a corner where I didn't belong.”*

As Beebeejaun (2017) argues, spatial design is never neutral; it is a concrete enactment of gendered power relations. The naming, division, and allocation of space function as techniques of discipline. Spade (2015) further highlights how administrative violence, grounded in gender classification, does not rely on overt hostility but instead operates through the systematic management of identity information, making it exceedingly difficult for transgender individuals to obtain a stable and visible place within institutional structures. When the question “Where do you belong?” is reduced to a binary institutional demand, my experience becomes one not of freedom to choose, but of being structurally limited in my options.

#### 3.2.3 Bodily withdrawal

This logic of exclusion extends into the bodily routines of daily life. I began to deliberately reduce my fluid intake to avoid the discomfort and anxiety of navigating restroom access. After class, I often left in haste, unable to find a suitable space to clean my body. While the campus did include gender-inclusive restrooms, they were few and located far from teaching facilities, rendering them impractical for everyday use. These reflections were consistently coded under “bodily withdrawal,” a sub-theme that connected individual coping strategies (e.g., reduced fluid intake, leaving facilities early) to the broader analytic theme of institutional spatial discipline. *As I recorded in my journal: “I would rather stay thirsty than risk the walk to a restroom where I know I don't belong.”* When the institution fails to formally recognize the legitimacy of transgender identities, and the body has not yet undergone full transition, these small but frequent decisions become internalized as bodily restraint and spatial withdrawal.

Doan (2010) observes that institutions not only determine who is visible but also define who has the right to appear in space and in what form. When I hesitate at the bathroom door, avert my gaze at the side entrance of the swimming pool, or leave the gym early because I could not shower like others, each of these ordinary actions becomes a testament to my effort to persist within institutional space. Ahmed (2012) reminds us that “institutional silence is not an absence; it is a selective unwillingness to see.” Such a refusal to see is not merely a denial of personhood, but a cancellation of existence itself.

#### 3.2.4 Summary

Thus, the narratives in this section illustrate how “gendered spatial exclusion” emerged as a major theme, showing how architectural design and tacit administrative practices force transgender teachers into constant silent negotiation. By making explicit the analytic coding process, the link between lived experience and thematic interpretation becomes transparent.

As a teacher, I have been permitted entry into the classroom. But as a transgender person, I have never been given a space in which to fully exist. This is not simply a question of where to shower, change clothes, or use the restroom, it is a fundamental inquiry into whether the institution recognizes a person. When every spatial trajectory is pre-coded with gendered assumptions, and I cannot locate myself within them, what appears to be neutral campus architecture becomes, in fact, one of the most powerful enactments of gendered discipline.

### 3.3 Teaching in tension: neither a male nor a female teacher

Within Taiwan's educational system, although teacher-student interactions often utilize the ostensibly gender-neutral term laoshi (teacher), the logic of gender classification remains deeply embedded. Compared to educational cultures in Hong Kong or Anglophone countries where gender-marked titles such as Sir or Miss are commonly used spoken references in Taiwan appear more uniform and ambiguous, suggesting a neutral linguistic context in the classroom. However, this surface-level gender neutrality often paradoxically reinforces grammatical and social norms of gender recognition in practice.

Written language and administrative governance require the explicit distinction between “he” and “she,” a distinction that functions both as a grammatical convention and as a tool of institutional recognition. This convergence of linguistic normativity and institutional identification renders a teacher's gender not merely biographical, but structurally integral to pedagogical authority and classroom order (Butler, 2002). In highly embodied teaching contexts such as physical education, the teacher's gender identity becomes an unspoken yet pivotal rule governing course operations, authority construction, and professional legitimacy.

In the coding process, narratives like these were grouped under the theme “teaching in tension,” which captured the constant negotiation between linguistic categories, institutional expectations, and embodied classroom practices. Sub-codes such as “role ambiguity,” “student expectations,” and “pedagogical adaptation” emerged directly from reflective journal entries and were clustered to reveal the multi-layered pressures shaping the teaching persona of transgender educators.

#### 3.3.1 Role ambiguity

For transgender teachers, navigating this linguistically and institutionally constructed gender regime is not simply a matter of expressing one's identity; it is a daily exercise in negotiation and adaptation. As a transgender educator, I often find myself caught between students' gendered perceptions and institutional misclassification. While most students regard me as a “male teacher,” they are frequently puzzled or unsettled when I fail to exhibit stereotypically masculine behaviors such as commanding voice projection, authoritative presence, or assertive speech. Simultaneously, I am excluded from certain cultural privileges often afforded to female teachers, such as students voluntarily helping with equipment or perceiving the teacher as someone to be protected in moments of classroom disorder. *In one reflection, I wrote: “Too soft, and I am not a real man; too strict, and I am not the kind of female teacher they imagine—there is no role I can safely occupy.”*

This situation reflects my inability to fully inhabit any pre-scripted gender role Schilt and Westbrook, (2009), leaving my teaching persona suspended in a state of ambiguity and fluctuation. In thematic coding, these accounts were consistently coded under “role ambiguity,” which highlighted how reflective notes about student reactions and institutional gender expectations translated into an analytic category that connects the personal narrative to broader discussions of gender performativity.

#### 3.3.2 Student expectations

The physical intimacy inherent in PE classes, individual coaching, demonstrating movements, and unavoidable body contact places me in a heightened state of self-regulation and risk assessment. Even when certain actions are pedagogically necessary and professionally appropriate, I must continually evaluate the potential for student misunderstanding and adjust, defer, or reconfigure my teaching strategies accordingly. *As I noted in my journal: “Every time I correct a student's posture, I rehearse the move in my head first, asking myself if it could be misread.” Another entry reads: “What should feel like a simple teaching touch often feels like stepping into a spotlight where any gesture can be questioned.”*

#### 3.3.3 Pedagogical adaptation

One concrete strategy I have developed in response to these challenges is to address issues of bodily interaction explicitly at the very beginning of class. I emphasize to students that physical contact is not permitted simply on the basis of being of the same gender; rather, professional boundaries must be respected regardless of gender identity. This initial clarification not only serves as a form of self-protection, but also functions as an ethical and pedagogical intervention. Drawing on my parallel role as an instructor of fitness coaching, I integrate relevant legal frameworks and disciplinary knowledge to explain how professional trainers should avoid behaviors that could be construed as sexual harassment. By situating these guidelines within broader discussions of gender diversity in sports, I seek to transform what might otherwise be perceived as a private vulnerability into a shared pedagogical moment—one that equips university students with essential literacy in both professional ethics and gender inclusivity.

*In one reflection, I wrote: “Starting every class with clear rules is not just for them, it is for me—to remind myself I can claim authority without pretending to be someone else.” Another entry notes: “When I frame boundaries as professional ethics, students stop seeing me as ‘different' and start seeing me as a competent teacher.”*

These classroom strategies were categorized in the sub-code “pedagogical adaptation,” illustrating how reflective entries documenting my proactive interventions (e.g., boundary-setting, ethics framing) were analytically elevated into evidence of how transgender teachers transform vulnerability into professional strength.

As Airton (2009) emphasizes, transgender teachers must constantly assess the social legibility and acceptability of their gender presentation in every pedagogical interaction, while simultaneously considering whether such performances may threaten their physical safety or professional credibility. Physical education, as a highly embodied profession, turns the teacher's body not only into a medium for knowledge transmission but also into a site where gender meanings are persistently scrutinized, interpreted, and contested.

Within this intersection of power and language, I am compelled to maintain a contradictory dual posture: on the one hand, upholding pedagogical principles and professionalism; on the other, incessantly self-monitoring to avoid behaviors that could be read through a gendered lens. In this hyper-regulated teaching environment, gender is no longer a background identity marker but infuses every posture, every proximity to students, and every word I choose. Butler's (2002) concept of gender performativity materializes here not as a voluntary expression, but as an embodied strategy of constraint: I do not perform gender freely, I strategically modulate my gendered presence to avoid being “misread.”

#### 3.3.4 Summary

Thus, the theme “teaching in tension” synthesizes these coded patterns—role ambiguity, student expectations, and pedagogical adaptation—into a coherent analytic category. By explicitly linking narrative fragments to thematic coding, the analysis demonstrates how lived experiences are systematically interpreted into broader conceptual insights about professionalism, authority, and gender performativity in education.

This ongoing modulation and suppression compel me to rethink what it means to be professional. Professionalism is no longer confined to content mastery or pedagogical delivery, but extends to the real-time management of gender expectations, student perceptions, and institutional norms. In this process, what I experience is not simply a teaching challenge, but a profound confrontation with, and redefinition of, normative gender structures.

### 3.4 Sexual harassment and powerlessness

In the thematic analysis, such incidents were consistently coded under the category “sexual harassment and institutional silence.” Sub-codes included “student provocation,” “fear of misinterpretation,” and “absence of reporting channels.” These codes, drawn directly from reflective notes of classroom encounters, were clustered to illustrate how embodied harassment was compounded by structural invisibility, producing a unique form of professional vulnerability for transgender teachers. This section draws on Spade's (2015) theory of administrative violence and Ahmed's (2012) concept of institutional silencing to analyze how transgender teachers face structural vulnerability when encountering sexual harassment. These narratives demonstrate not only the embodied nature of harassment in physical education settings but also the absence of institutional mechanisms to acknowledge transgender teachers as legitimate victims.

Early in my career as a part-time PE teacher, I had not yet legally changed my gender marker nor publicly disclosed my transgender identity. This “in-between” status placed me outside the institutional binary, amplifying my sense of precarity. Physical education, with its reliance on proximity, shared spaces, and bodily demonstration, intensified the risk that my actions could be misinterpreted or stigmatized. As Jones et al. (2014) note, when institutions lack explicit protections for gender identity, teachers are preemptively positioned as potential risks rather than individuals in need of safeguards.

#### 3.4.1 Student provocation

On one occasion, two female students provocatively invited me to engage in a “3P” (threesome) during class, while a male student deliberately made physical contact with me. Although I experienced clear discomfort and emotional shock, what disturbed me more deeply was the realization that within a system that assumes teachers to be “potential perpetrators” (Christensen and Darling, 2020), any assertive reaction I made could subject me to investigation or media scandal framed as a “transgender teacher controversy.” *In my reflective notes, I wrote: “Their words stung, but what terrified me most was knowing that if I resisted, I could be the one put on trial.”*

#### 3.4.2 Fear of misinterpretation

This context left me unable to report the incident or file a complaint. As a non-tenured instructor, I lacked union protection, and the existing gender equity procedures offered no mechanism for individuals with ambiguous legal or gender status to initiate a formal claim.

In the thematic analysis, such incidents were consistently coded under the category “sexual harassment and institutional silence.” Sub-codes included “student provocation,” “fear of misinterpretation,” and “absence of reporting channels.” These codes, drawn directly from reflective notes of classroom encounters, were clustered to illustrate how embodied harassment was compounded by structural invisibility, producing a unique form of professional vulnerability for transgender teachers.

This experience illustrates what Spade (2015) identifies as administrative violence: not violence born of direct hostility, but rather of rules, classifications, and bureaucratic language that render certain subjects institutionally unrecognizable. Ahmed (2012) further argues that when institutions fail to acknowledge identities, those individuals are denied the capacity to articulate grievances and be heard. For me, this constituted a form of institutional silencing (Tiitinen, 2020): I could not be recognized as a “victim,” nor could I legitimately “speak,” and thus silence became the only viable survival strategy to preserve my job. *As I noted in my journal: “Every word I held back felt like a shield—I stayed silent not because I wanted to, but because any speech could be turned against me.”*

#### 3.4.3 Absence of reporting channels

These reflections were also coded as “silence as survival,” a theme that captured how repeated journal entries described withholding speech not as a free choice but as a structural imposition. This analytic link demonstrates how the narrative data were translated into the broader theoretical insight that silence itself is an institutional outcome rather than an individual coping preference.

The hostile media environment further reinforced this silence. As Koshkarova et al. (2019) notes, mainstream media often misrepresents transgender issues through biased narratives and non-neutral framings, leading to stigmatization in the public sphere. I knew I had experienced injustice, but I also understood that any act of speaking out would likely result in personal consequences and potentially shape public opinion against transgender teachers more broadly. *As I reflected in my notes: “Even if I wanted to file a complaint, where would I go? There was no office, no form, no channel that could even acknowledge my existence.”*

Empirical research has shown that gender equality policies in educational institutions are frequently symbolic. Timmers et al. (2010), in their analysis of gender policies across 14 Dutch universities, found that while policies addressed cultural, personal, and structural dimensions, their actual implementation was highly dependent on departmental support and managerial will, often lacking effective monitoring or accountability mechanisms. In Taiwan's higher education system, transgender teachers are, in theory, protected under the Gender Equity Education Act and the Employment Services Act. In practice, however, many still encounter systemic barriers such as mismatches between gender markers and lived appearance (Currah and Mulqueen, 2011; James et al., 2016), as well as restricted access to gendered spaces (Seelman, 2016). These challenges often force teachers to conceal their identities to maintain employment security. Such conditions reflect what Timmers et al. (2010) describe as “symbolic policies” superficial frameworks that fail to incorporate the lived realities of gender-diverse individuals or provide actionable mechanisms for redress and protection.

#### 3.4.4 Summary

In short, I was not unhurt, I was simply unrecognizable as a “victim” within the existing institutional framework. When I was neither protected by policy nor able to bear the risks of speaking out, silence became the only viable tactic. But this was not a choice; it was the result of being structurally positioned outside the boundaries of institutional legibility and legitimacy.

Thus, the theme “sexual harassment and powerlessness” synthesizes these coded narratives into a broader analytic claim: that harassment cannot be separated from institutional misrecognition, and that the inability to speak or report is itself an outcome of administrative and epistemic violence. By explicitly linking narrative episodes to their thematic coding, the analysis demonstrates how individual experiences provide systematic evidence for theorizing the intersections of harassment, silence, and professional precarity in transgender teachers' lives.

### 3.5 Negotiating self-identity and professional role

As a transgender teacher, my pedagogical journey has never followed a linear or smooth trajectory. Especially during my initial entry into the educational field, I encountered student provocations involving sexual innuendo and unsolicited physical contact. My response was not only anger and shock but a deeper anxiety about whether I could truly “belong” in such a professional environment. At that time, I was neither legally recognized as a male teacher nor institutionally affirmed in my professional identity. The sense of vulnerability I experienced did not stem solely from isolated incidents, but from a more pervasive structural insecurity about my future in the profession.

In thematic analysis, these journal entries were coded under the theme “negotiating identity and professionalism,” with sub-codes such as “self-silencing,” “gendered performance,” and “pedagogical adaptation.” This analytic framing allowed personal crises of belonging to be systematically linked to the broader challenge of reconciling gender identity with professional legitimacy.

#### 3.5.1 Self-silencing

These experiences are not uncommon for transgender educators. Antonelli and Sembiante (2022) note that many LGBTQ+ teachers adopt self-silencing strategies to suppress gender expression to avoid discrimination, complaints, or stigmatization and to maintain job security. Before I had built a sufficient support system or developed a stable sense of professional confidence, silence became my only available survival strategy. For an entire month, I worked through trauma and emotional exhaustion, simply to determine whether I still had the legitimacy to stand in front of a classroom whether I still had the right to “be a teacher.” *As I wrote in my journal: “Every morning I asked myself—can I survive another day without speaking who I am?”*

#### 3.5.2 Pedagogical adaptation

Yet it was precisely within this space of rupture and uncertainty that I began to realize the necessity of engaging in a deliberate process of negotiating between self-identity and professional practice. Rather than investing my energy in suppressing myself, I began to consciously adapt my classroom strategies modifying clothing choices, vocal tone, and power dynamics in teacher-student interaction to construct a teaching persona that was not confined by traditional gender roles, but still capable of asserting classroom authority. These reflective accounts were coded as “pedagogical adaptation,” highlighting how shifts in voice, clothing, and authority style became recurring strategies to navigate institutional expectations. This was not merely a matter of avoiding conflict; it was a shift toward practicing gendered ethics what Boler (2004) describes as an approach in which teachers reject blind conformity to social gender norms and instead co-construct ethical learning environments in dialogical interaction with students. *In my notes, I wrote: “Each adjustment—how I dress, how I speak—became less about hiding and more about creating a way to teach on my own terms.”*

#### 3.5.3 Gendered performance

In the beginning, I mistakenly believed that to be recognized as a “male teacher,” I had to perform masculinity through outward appearance and vocal delivery. At times, this meant deliberately wearing a suit in formal school contexts or adopting an excessively forceful tone in class in order to assert authority and confirm my male identity. Over time, however, I came to realize that such overemphasis on gendered performance not only felt unsustainable but also narrowed the pedagogical space available for building authentic relationships with students. *As I reflected in my journal: “The suit gave me authority, but it also built a wall between me and the students.”*

Gradually, I shifted toward a more neutral and professional style: in terms of clothing, I chose practical athletic attire that underscored my expertise as a physical education instructor rather than signaling gender; in terms of voice, I avoided either artificially deepening or softening my tone, instead maintaining clarity, stability, and a balance of authority and approachability. These shifts allowed me to redirect students' focus away from my gender and toward the substance of my teaching. Analytically, these shifts were clustered under “professional coherence,” a sub-code that linked identity negotiation with the consolidation of pedagogical authority.

#### 3.5.4 Student validation

Amid this transformation, I began to receive informal yet meaningful feedback from students and colleagues. Some students told me, “You're gentler than other teachers, so I feel more comfortable asking questions,” or “You're not as harsh as other PE teachers, I prefer this kind of class.” These seemingly minor comments became critical sources of validation, especially during moments when I was in greatest need of reassurance. *As I wrote in my notes: “Their small words felt like lifelines—proof that I could be accepted not despite my difference, but through my way of teaching.”*

As Brant and Willox (2021) argue, positive student feedback can serve as symbolic allyship for gender-nonconforming educators, mitigating institutional alienation, and reinforcing their teaching self-efficacy.

More importantly, these interactions revealed that gender was no longer the central metric by which students assessed my teaching. Some students even admitted they had sensed something “different” about me but chose to disregard the label because they appreciated my pedagogical style and course content. Such feedback was coded as “student validation,” which in the analysis linked lived experiences of recognition to the broader theme of professional legitimacy. This led me to a deeper understanding: if my teaching persona was coherent, consistent, and sincere, students possessed the cognitive flexibility and emotional maturity to accept a gender-nonconforming professional. This resonates with Britzman's (1998) assertion that the teacher's professional identity is not a passive replication of social norms, but the emergent product of ongoing negotiation with students, culture, and institutional forces.

#### 3.5.5 Summary

This process ultimately enabled me to reframe my transgender experience as a form of critical capital within pedagogical practice. I no longer viewed being transgender as a constraint, but as a productive site for re-asking fundamental questions: “Who gets to be a teacher? What can a teacher look like?” As Hooks (2014) asserts, “The margin is not a site of rejection, but a location of radical possibility a space for reflection and reconstruction of knowledge and power.” I began to believe that if I continued to teach with passion and integrity, even the disclosure of my gender identity would no longer constitute a threat. Rather, it empowered me to more fully inhabit a teacher identity grounded in difference as pedagogical value—a teacher who helps students see that difference itself can be a source of strength and insight.

Thus, the theme “negotiating identity and professionalism” synthesized these coded elements—self-silencing, pedagogical adaptation, gendered performance, professional coherence, and student validation—into a broader analytic insight: that transgender identity, when reflexively negotiated, can become a source of pedagogical strength and inclusive practice.

## 4 Discussion

This study, through autoethnography and embodied experiences in educational settings, reveals how transgender teachers, during their gender transition, encounter intersecting pressures of structural exclusion and cultural dissonance within administrative systems, campus spaces, and pedagogical practices. These experiences are not merely personal narratives but also reflections of deeper institutional and cultural misalignments in Taiwan's gender governance within education. This discussion is structured around three key dimensions: (1) the rupture between administrative systems and cultural recognition; (2) the destabilizing effects of gender ambiguity on school order; and (3) the dual nature of transgender identity as both constraint and pedagogical resource.

### 4.1 Administrative-cultural disjunction: when legal progress fails to penetrate institutional inertia

Although Taiwan legalized same-sex marriage in 2019 and has increasingly adopted inclusive gender language in legal discourse, educational administrative systems continue to operate under rigid binary gender logic. This gap between legal progress and institutional practice (Ahmed, 2012) becomes evident in routine procedures such as faculty sign-ins, public announcements, and roster registrations, where transgender teachers frequently encounter misgendering. These routine tasks often result in involuntary outings and institutional exposure (Spade, 2015). What appears to be administrative routine in fact becomes an exclusionary classificatory technique that denies non-normative gender identities institutional legibility as theorized by Spade.

This exclusionary logic also extends into off-campus educational settings. For instance, when serving as an instructor at a senior care center, my teaching was highly praised; however, once ID documentation was requested for administrative processing, the discrepancy between my gender marker and appearance led to the abrupt termination of the collaboration. Building on this, Hazeldean (2019) further illustrates how an insistence on uniform data records transforms official documents from neutral identifiers into mechanisms of exclusion. Such reliance on bureaucratic consistency exposes the lack of institutional frameworks that could otherwise accommodate gender diversity, thereby revealing a cultural deficit in Taiwan's administrative structures.

Moreover, the intersection of gender and social class further intensifies these barriers. In Taiwan, more affluent transgender individuals may pursue legal remedies such as administrative litigation to change their gender markers without undergoing surgery. For example, on May 30, 2024, a transgender man known as Nemo successfully changed his legal gender through an administrative court ruling. By contrast, those without sufficient financial resources are often left with no viable options, as gender-affirming surgeries are largely self-funded in Taiwan, and legal gender recognition typically remains contingent on such costly medical interventions. This disparity demonstrates how economic inequality compounds the challenges of navigating administrative and cultural exclusion.

### 4.2 Gender ambiguity as institutional challenge and pedagogical dilemma

The mere presence of transgender teachers disrupts the binary gender order of the school. As Doan (2010) asserts, space is never neutral, it is a site of gendered power. This study reveals that spaces such as swimming pools, gyms, and gender-segregated restrooms become contested terrains for transgender educators. Even when social appearance and interpersonal interaction align with a male identity, lack of administrative gender recognition restricts access, forcing individuals into marginal “exception spaces” (Browne, 2004). This is not only a matter of spatial logistics but also an institutional denial of gendered existence.

Such “exception spaces” are particularly harsh for teachers with limited resources, since social class shapes whether one can seek alternative work environments or purchase access to private facilities, thereby producing a dual exclusion of both gender and class. In the Taiwanese context, this divide is also reflected in higher education institutions. Faculty positions at elite national universities are widely recognized as more prestigious and better resourced, with comparatively greater access to gender-inclusive facilities such as all-gender restrooms. By contrast, faculty at lower-ranked private universities—including the first author of this study—often work under 1-year renewable contracts with limited institutional authority and minimal job security. Within such precarious conditions, advocating for gender-inclusive facilities is nearly impossible, as voicing such concerns may jeopardize even the basic opportunity to retain employment. This illustrates how classed hierarchies within academia intersect with gender non-conformity to further marginalize transgender educators.

On the level of classroom interaction, occupying a position outside “typical male” or “typical female” identities creates ambiguities in pedagogical authority. Transgender teachers often expend additional emotional labor to establish classroom control. While such non-normative gender performances may be interpreted as subversive acts that challenge hegemonic gender norms (Butler, 2002), in practice they often translate into professional vulnerability and increased labor burdens (Airton, 2013). Gender ambiguity thus emerges not only as a cultural disruption but as an institutional problem that existing systems are ill-equipped to address.

### 4.3 Transgender identity as both limitation and transformative resource in education

Despite the structural precarity and exclusion faced by transgender teachers, this study also illustrates the transformative potential of transgender identity when pedagogical trust and classroom management are gradually established. Positive student feedback regarding the teacher's gentle interaction style suggests that gender difference is not a barrier to teaching efficacy; rather, it can foster more democratic, trust-based learning environments (Brant and Willox, 2021).

These findings highlight that transgender teachers are not merely subjects of institutional conflict but can also act as critical agents for pedagogical innovation and institutional reflection. As Hooks (2014) reminds us, marginality should not only be seen as a site of rejection but as a space for knowledge production and the reconfiguration of power. Through their lived experiences, transgender teachers are positioned to question cultural assumptions such as “who can be a teacher” and “what should a teacher look like,” thereby challenging gendered norms of professional identity (Britzman, 1998).

A relevant comparative study is Suárez's (2022) autoethnography, which centers on a transgender teacher's journey to claim an authentic teaching self through narrative reflection. By positioning the classroom as both a site of personal negotiation and social intervention, Suárez demonstrates how transgender educators can reframe marginality into pedagogical strength. Complementing this, Green et al. (2024) present a co-authored autoethnography in mathematics teacher education (a genderqueer preservice teacher with a cis male educator) showing that coming out is a continuing process, trust is co-constructed, and unacknowledged misgendering inflicts tangible harm; they also translate these insights into practice (e.g., explicit rationales for pronoun sharing, private disclosure channels, and audits of gendered language/materials). Together, (Suárez 2022) and (Green et al. 2024) provide methodological and practical precedents that sharpen this study's focus on the transformative agency of transgender educators within structurally constraining environments.

Yet this creative potential at the margins is itself stratified by social class. Teachers with greater cultural capital and financial resources are often better positioned to convert non-normative gender identities into professional advantages, while those from working-class or economically disadvantaged backgrounds face limited opportunities to do so, owing to the lack of institutional protections and professional networks. In Taiwan, this disparity is especially pronounced because gender-affirming treatments and surgeries are largely self-funded; those with means can navigate legal recognition and medical transitions more quickly, whereas those without sufficient resources may struggle even to afford the repeated medical consultations required for pre-surgical assessments. By contrast, in Hong Kong, although the waiting periods for transgender-related healthcare are lengthy, medical consultations and surgeries are partially covered under public healthcare, reducing the extent to which financial resources determine access. This comparison underscores how class and national healthcare systems jointly shape the capacity of transgender teachers to transform marginalization into pedagogical and professional resources.

These findings highlight a critical gap in teacher education: while considerable emphasis is placed on teaching diverse student populations, the needs of transgender educators remain largely overlooked (Airton, 2019). Most programs continue to presume cisnormative identities and offer little to no preparation for addressing legal ambiguities, institutional misrecognition, or gendered classroom dynamics (Wernick et al., 2017). Without adequate support, transgender educators are left to navigate these challenges on their own. To foster genuine inclusivity, teacher training must go beyond formalistic approaches and incorporate modules on administrative negotiation, classroom authority, and advocacy for gender-inclusive policies (Kosciw et al., 2020). Such reforms are essential not only to affirm transgender teachers as professionals, but also to recognize them as agents of change within educational systems.

However, such transformative possibilities do not imply systemic protection. When transgender teachers face sexual harassment or inappropriate interactions from students, their lack of legal recognition often prevents them from accessing institutional mechanisms designed to address such issues (Timmers et al., 2010). The absence of procedural sensitivity and institutional responsiveness renders these educators vulnerable, forcing them to navigate between self-protection and professional risk. This reflects what Pinder and Harlos (2001) call institutional silence not simply a policy gap, but a systemic refusal to see and support certain identities.

## 5 Conclusion

This study, through an autoethnographic approach, offers an in-depth account of a transgender teacher's negotiation of identity and institutional constraints during the process of gender transition within the educational field. The findings reveal that despite growing awareness of gender equity, educational administration, and institutional culture remain deeply rooted in binary gender assumptions. As a result, transgender educators frequently encounter systemic misalignment and exclusion in spatial arrangements, identity documentation, employment procedures, and classroom interactions. Particularly in cases where legal gender recognition has not yet been granted, teachers exist in a liminal zone, lacking institutional acknowledgment, and legitimate grounds for action leading to persistent tension between professional roles and personal identities.

Nonetheless, the study also demonstrates that transgender teachers are not merely passive recipients of institutional violence. On the contrary, they actively construct alternative professional identities through pedagogical language, embodied practice, and affective engagement. These strategies though non-normative prove persuasive and effective, garnering recognition, and support from students and peers. Such agency not only illustrates the transformative capacity of transgender educators within schooling contexts but also challenges normative assumptions about who is entitled to occupy the role of “teacher.”

From a practical standpoint, this study offers several key recommendations for future educational reform. First, administrative processes should incorporate flexible gender options to avoid unnecessary disclosure and identity-based oppression. Second, gendered spatial arrangements in schools—particularly restrooms and changing facilities—should be reconfigured to promote inclusivity and spatial justice. Third, experiences of harassment directed toward gender minority teachers should not be left unaddressed by institutions. Schools must establish accountable anti-harassment policies and response mechanisms to interrupt the pattern of administrative silence justified by claims of uncertainty or lack of procedure. Even in non-hostile environments, such inaction can reinforce structural marginalization. Fourth, teacher education programs should include training modules on gender diversity and field-based response strategies, equipping future educators with practical tools to recognize and intervene in incidents of exclusion or harassment.

Finally, this study acknowledges its limitations. As an in-depth single-case narrative, it provides rich insights into institutional mechanisms and individual experiences; however, further research is necessary to develop a broader theoretical and policy framework. The conditions described in this study—such as binary-based institutional design, lack of gender recognition mechanisms, and the emotional labor required for pedagogical legitimacy—are not unique to Taiwan. Educational systems across East Asia often exhibit similar structural characteristics, including rigid gender norms, bureaucratic inertia, and insufficient teacher training on gender diversity. Therefore, the insights derived from this autoethnography may hold relevance and resonance in other East Asian educational contexts that face comparable gaps in trans-inclusive policy and practice. Future investigations should consider cross-cultural comparison, mixed methods design, and intersectional analyses—particularly those exploring the interrelation of gender identity and other marginalized identities such as disability—to further expand the scope of gender governance research in education.

While this study focuses on the author's experience as a transgender teacher whose appearance is predominantly read as male, future research should critically engage with the experiences of educators whose gender embodiment and expression resist binary categorization. Non-binary and gender non-conforming teachers often inhabit a position of heightened “unreadability” within institutional frameworks, where their bodies and identities cannot be easily assimilated into existing classificatory systems. This liminal status not only intensifies administrative and spatial exclusion but also unsettles normative assumptions of professional authority and pedagogical credibility. By examining how non-binary educators negotiate recognition, manage affective labor, and construct legitimacy in contexts that fail to account for them, future scholarship can move beyond the binary logic of gender governance and contribute to more nuanced theories of institutional diversity. Such research is crucial for developing educational policies and pedagogical practices that acknowledge the full spectrum of gendered subjectivities and respond to the increasing visibility of non-binary teachers in contemporary schooling.

## Data Availability

The original contributions presented in the study are included in the article/supplementary material, further inquiries can be directed to the corresponding authors.
